# Studying social accountability in the context of health system strengthening: innovations and considerations for future work

**DOI:** 10.1186/s12961-019-0438-x

**Published:** 2019-03-29

**Authors:** Victoria Boydell, Heather McMullen, Joanna Cordero, Petrus Steyn, James Kiare

**Affiliations:** 1 Global Health Centre, Geneva Institute of International and Development Studies, Geneva, Switzerland; 2 Queen Mary, University of London, London, United Kingdom; 3 Human Reproduction Program, World Health Organization, Geneva, Switzerland

**Keywords:** Social accountability, maternal health, sexual and reproductive health, study design, methodology

## Abstract

There is a growing body of research on the role of social accountability in bringing about more accessible and better-quality healthcare. Here, we refer to social accountability as “*citizens’ efforts at ongoing meaningful collective engagement with public institutions for accountability in the provision of public goods*” (Joshi, *World Dev* 99:160–172, 2017). These processes have multiple interrelated components and sub-processes and engage a range of actors in community-driven, often unpredictable and context-dependent actions, which pose many methodological challenges for researchers. In June 2017, scientists and implementers working in this area came together to share experiences, discuss approaches, identify research gaps and consider directions for future studies. This paper shares learnings from this discussion.

In particular, participants considered (1) how best to define and measure the complex processual nature of social accountability; (2) the study of social accountability as an inherently political process; and (3) the challenges of generalising unpredictable, community-driven and context-dependent processes. Key among a range of consensus areas was the need for researchers to capture a broader range of outcomes and better understand the nuances of implementation processes in order to effectively test theories and assumptions. Furthermore, power relationships are inherent in social accountability and the research process itself. In presenting details on these deliberations, we hope to prompt a wider discussion on the study of social accountability in health programming.

## Introduction

Social accountability is gaining rapid acceptance as a way to address health systems inefficiencies and improve basic public health performance, including planning and service delivery, and to contribute to the attainment of the highest possible standards of health. Here, we refer to social accountability as “*citizens’ efforts at ongoing meaningful collective engagement with public institutions for accountability in the provision of public goods* ([1], p. 161).”^1^ For example, the use of community scorecard processes to improve maternal health services, whereby health providers and community members assess their existing entitlements in service delivery and then prioritise the issues they face in accessing and delivering services [2, 3]. These priorities are then jointly shared in multi-stakeholder meetings with health officials, health facilities and the community to identify local solutions and actions for service improvement and to promote mutual responsibility and accountability. Other social accountability processes use a range of tools to trigger such changes, including social audits, public expenditure tracking systems, information campaigns, public hearings, participatory budgeting, and social movement to name a few. Interest in accountability has spurred a proliferation of studies on social accountability and other participatory processes in the context of health programmes. Yet, there has been less discussion on how to go about studying these dynamic and complex change processes.

The evidence on social accountability and health is growing fast. Early reviews found mixed evidence for the approach, partly due to the varied definitions of accountability used and the range of studies undertaken [4, 5]. Another set of reviews summarised the quantifiable benefits of social accountability for service improvement, service utilisation and health outcomes using more linear cause–effect measures [4, 6–9].

The definition of success encompasses more than directly measurable health-related outcomes and includes a wider range of governance outcomes such as empowerment, participation, and the responsiveness of duty-bearers [10–17]. Joshi notes the expanding number of outcomes related to social accountability “*are expected to unfold, and range from immediate short-term improvements in public services, to more durable long-term changes in states and societies*” ([1], p. 162). Evaluating a broader range of interrelated outcomes poses several methodological challenges.

In response to these methodological challenges, recent studies on social accountability in health service delivery have attempted to document and assess the complexity of activities and outcomes. Among the methodologies or mixes of methods used are randomised control trials to measure the impact on health and service delivery outcomes [2, 3], realist evaluations of social accountability programming [18, 19], process evaluations of the implementation processes [20], psychometric measures of governance outcomes [17], and Most Significant Change approaches to trace change [21, 22], all of which are applied retrospectively and prospectively. These approaches have been developed independently and present a wealth of innovation and experience in their conceptualisation, design and execution. There is, therefore, much to be learned from existing research practice.

In June 2017, researchers and implementers had the opportunity to share experiences, methodologies and findings to help guide future endeavours. WHO’s Department of Reproductive Health and Research, which includes the UNDP/UNFPA/UNICEF/WHO/World Bank Special Programme of Research, Development and Research Training in Human Reproduction, convened an event on studying social accountability in Geneva, Switzerland. In this forum, researchers and implementers of social accountability in the context of health, and specifically in reproductive, maternal, newborn and child health, shared experiences, challenges and successes (see Acknowledgements section for list of participants). This paper shares learnings from this discussion.

In particular, participants considered (1) how best to define and measure the complex processual nature of social accountability; (2) the study of social accountability as an inherently political process; and (3) the challenges of generalising unpredictable, community-driven and context-dependent processes.

### Defining and measuring the complex processual nature of social accountability

Social accountability processes aim to support service users voice their needs and make claims to their entitlements and hold those responsible for the provision of quality services to account. Accountability processes feature multiple and interrelated components, steps and actors, with several simultaneous processes of change, triggering collective changes. This process adheres to the definition of a complex intervention [23, 24]. Therefore, the object of study is a highly unpredictable process with a range of outcomes, and it accordingly requires some methodological innovation. Difficulties in defining the boundaries of the intervention and outcomes of interest can prove challenging in the conceptualisation of research and the development of appropriate study designs. Given this complexity, the study of social accountability interventions can risk either being reduced to mechanistic formulas or dismissed as too context-specific and lacking a generalisable value that would allow replication.

One challenge is that social accountability is relational and focuses on the relationship between rights-holders and duty-bearers; therefore, interventions change and adapt as they are rolled out in specific localities and relationships [25]. This unpredictability may lead to activities and strategies falling out of line with pre-set interventions. To address this, many researchers described ways they documented and examined implementation to better accommodate adaptive interventions. Process evaluations are increasingly being used to effectively explore fidelity, dose, reach and cost, and realist evaluation methodologies have also been used to assess fidelity, context, mechanisms, and provide lessons for replication and scale-up [18].

Descriptive and analytic work on implementation is further complicated by the broad and interrelated set of outcomes that together facilitate change. Outcomes may include both routine health and service delivery outcomes, alongside changes in self-efficacy, social cohesion, trust and responsiveness. Less conventional outcomes in public health, such as increased responsiveness of service providers and policy-makers or the emergence of new norms around empowerment of community members and community capabilities [18, 26], pose their own challenges because the metrics used to capture them are not standardised or widely accepted. Relatively new outcomes of interest need formative work to distil the mechanisms of effect and the most appropriate way to measure them. There have been efforts to capture the newer constructs (e.g. empowering service providers, mutual responsibility, increased confidence of service users) using psychometric measures and structured observations, which have been tested and are now moving into multi-country validation [2, 14, 20]. Others have been using approaches such as the Bellwether approach, outcome mapping and process tracing to assess changes in duty-bearers’ actions as a consequence of such interventions [3, 27, 28]. These innovations can be learnt from and built upon.

The composite nature of complex interventions presents yet more methodological challenges. Even when the outcomes are delineated, it may be difficult to attribute them to a specific part of a multi-component intervention [24]. By their very nature, social accountability processes are not easily standardised in their design and delivery as they are highly influenced by local context. Fox’s reinterpretation of the evaluation of evidence moves beyond treating social accountability as a bounded, tactical intervention built for field experiments, toward social accountability as multi-pronged process that utilises multiple strategies to encourage collective action and voice [11]. This means that the pathways between intervention and outcome are not linear or easy to attribute, and that a mix of methodologies and a range of data sources may be required to sufficiently describe an intervention’s effects [24, 29]. Part of the challenge is determining whether the intervention was delivered as intended [30].

Evaluation strategies need to respond to this complexity and researchers are developing creative solutions. A recommended approach to studying complex interventions is to develop a theoretical understanding of the change process based on existing evidence and theory, and then model the complex intervention to inform the intervention and evaluation design [24, 29, 31]. Mixed methods approaches enable the collection of data which not only describes outcomes but also the processes, shedding light on factors affecting implementation, while still measuring key effects.

A range of tools and tactics are used to support social accountability processes, from community scorecards, audio-visual documentation of rights violations, village health committees and citizen charters. They may vary in focus, looking either broadly at health systems or focusing on specific service delivery points, and they vary in engagement type from collaborative problem solving to more adversarial approaches. These diverse social accountability processes share three broad components as a part of their theory of change, namely information, collective action and official response [5]. To date, elements of this theory of change have been evidenced, while others remain theoretical and need further work. Ideally, theories of change should be adaptive to the perceptions of participants and to changing conditions on the ground so that they are participatory and responsive.

Not only are social accountability processes unpredictable and the outcomes varied, but a host of activities, outcomes and ‘unintended outcomes’ (negative and positive) may not be recognised in many forms of data capture. Methodologies are needed to intentionally account for these; for example, the Most-Significant Change approach uses personal accounts of change to determine what is considered most significant, or diffusion of innovation research that studies the adoption of a practice among a group of stakeholders [21, 32]. Local social hierarchies and power dynamics influence the implementation of social accountability efforts. These are often unspoken, easily elude the most ‘robust’ study design and scuppering the tidiest field experiment design intervention, and speak to the value of qualitative research [33]. Ethnographic methods, such as long-term observation, can be helpful in capturing the implicit and taken-for-granted hierarchies of daily life [34].

### The study of social accountability as an inherently political process

The study of social accountability is never neutral as it is explicitly concerned with changing the power relationship between ‘citizens’ and the local duty-bearers, and are inherently political even if they do not intend to be. Many quantitative or clinical study designs attempt to control for and reduce complexity, which often means that the political dimensions of an intervention’s implementation are masked. These dimensions not only need to be recognised as germane to the trajectory and success of social accountability efforts, but also need to account for how the research process itself reflects or perpetuates power dynamics. Involving local political scientists in the evaluation processes can bring expertise and understanding of how power penetrates the capillaries of communities.

It is critical to acknowledge that those who actively challenge the authorities, even in research conditions, risk reprisals and social harm. The social, political and economic repercussions are substantial and, for many, disrespectful treatments may be pervasive and expected [11, 35]. Ethical research conduct should extend to these very real-life negative consequences for ‘research participants’ and requires the provision of safeguards and protections. For example, community-based organisations often set up a telephone hotline for supporters/participants when working on accountability. Fields such as anthropology and psychology commonly employ codes of practice (see the American Anthropologist Association’s Principles of Professional Responsibility and the American Psychological Association’s Ethical Principles of Psychologists and Code of Conduct). Given the political character and ethical considerations of studying social accountability, a similar code of conduct could be relevant.

Moreover, researchers could move beyond just accounting for power to examining their role in power relationships. This could entail supporting the communities that they work with, and addressing community members’ concerns and issues, even if this is by simply raising the issue with the relevant authorities, and even if those issues are beyond the study’s objective. There are always dynamics around who sets the research agenda – who decides what questions to ask, how to define impact and who is implicitly entitled to do this. There needs to be openness to the different roles that researchers, implementors and communities can play. More concrete examples of how and where this has been done in a way that considers interests of ‘researchers’, ‘implementers’ and communities affected by the issue/intervention/research, would be beneficial.

### The challenge of generalising unpredictable, community-driven and context-dependent interventions

The final area of consideration at the convening was on how best to discern generalisable findings from differently situated social accountability processes that adapt to local context. This area is relevant for replication, scale-up and sustaining successes after the intervention. There was consensus that, to assess an intervention’s potential for generalisability and replication, there needs to be consensus on which elements make for success. This involves unpacking the mechanisms of effect and determining what the ‘active ingredients’ are and how they relate to each other [23, 36].

Firstly, identifying scalable ‘active ingredients’ requires moving away from cause–effect models and towards mixed methods, which can relate thick qualitative descriptions with impact measures. As such, across the board, recent well-resourced projects have successfully built intervention monitoring and/or process evaluations into studies of impact. This has varied from better documenting the exact inputs for an intervention, to more detailed process evaluations, including extensive in-depth interviews and observations. Moreover, these processes do not happen in a vacuum but rather in real world settings, where other programmes and interventions may be operating in the same area along with uncontrollable factors external to the programme. Accordingly, many studies map out existing programming as well as other contextual factors (e.g. elections) at play using political economy analysis and process mapping [18, 37–39]. Contexts change and context analysis should capture context over time rather than a one-off activity that provides a snapshot.

Yet, even when we better understand ‘what works’, other factors may hinder these processes being replicated, scaled-up and sustained after the intervention. There are no guarantees that the stakeholders key in supporting any scale-up would be interested in doing so or have the appetite for scaling-up processes that challenge the status quo or authorities.

## Conclusion

To bring about accessible and better-quality healthcare, it is important to recognise both the value and complexity of social accountability interventions and operate accordingly. In doing so, we should test our theories and assumptions by building on what we know, capturing a broader range of outcomes, and accommodating unpredictable implementation processes. This work happens in the real world, with influential power relationships and high stakes for participants. By moving towards co-design and horizontal approaches, anticipating exclusion and ensuring that safeguards are in place to prevent and mitigate exclusion, the power inherent in the research process itself can change. Finally, we must acknowledge – and advocate – that this work takes time and requires context-specific adaptations.

## Footnotes

1 We recognise the limits of the word ‘citizen’, which excludes non-citizens who also have the right to make claims. We use the term ‘citizen’ as shorthand to refer to all rights-claiming people.
